# Effect of collaborative testing on learning and retention of course content in nursing students

**Published:** 2015-10

**Authors:** MOZHGAN RIVAZ, MARZIEH MOMENNASAB, PAYMANEH SHOKROLLAHI

**Affiliations:** 1 Student Research Committee, Department of Nursing ,School of Nursing and Midwifery, Shiraz University of Medical Sciences, Shiraz, Iran;; 2 Department of Nursing, School of Nursing and Midwifery, Shiraz University of Medical Sciences, Shiraz, Iran;; 3 Department of Nursing and Midwifery, Islamic Azad University, Firouzabad Branch, Iran

**Keywords:** Collaborative, Nursing education, Learning

## Abstract

**Introduction:**

Collaborative testing is a learning strategy that provides students with the opportunity to learn and practice collaboration. This study aimed to determine the effect of collaborative testing on test performance and retention of course content in nursing students of Shiraz University of Medical Sciences, Shiraz, Iran.

**Methods:**

This quasi-experimental study was carried out on 84 students enrolled in the course of Medical-Surgical 2 in Spring 2013 and Fall 2013 semesters. The control group consisting of 39 students participated in the first mid-term exam in an individual format. The intervention group, on the other hand, consisted of 45 students who took the test in a two-stage process. The first stage included an individual testing, while the second stage was a collaborative one given in groups of five individuals chosen randomly. Four weeks later, in order to investigate retention of the course content, both groups took part in the second mid-term exam held individually.

**Results:**

The study findings showed significant difference between the mean scores in the intervention group in the Fall 2013 semester (p=0.001). Besides, a statistically significant difference was found between the two groups regarding the tests mean scores (p=0.001). Moreover, retention of course content improved in the collaborative group (p=0.001).

**Conclusion:**

The results indicated an increase in test performance and a long-term learning enhancement in collaborative testing compared with the traditional method. Collaborative testing, as an active learning technique and a valuable assessment method, can help nursing instructors provide the alumni with strong problem-solving and critical thinking abilities at healthcare environments.

## Introduction

A primary goal of nursing education is application of acquired knowledge from educational environments to clinical environments and society towards desired health outcomes. Active learning, as an educational strategy, will lead to learning enhancement, problem-solving skills, and critical thinking which are all essential for transfer and application of this knowledge (1). The safety and well-being of patients all demand experienced nurses capable of analyzing and managing multidimensional patient needs. Meeting patients’ care needs is in fact a collaborative and teamwork approach by healthcare professionals (2). Active learning means that students have more responsibility for their own learning and are more engaged in educational processes. Different active learning methods will help students expand concepts and apply the knowledge at clinical environments (3).

Collaborative testing is one learning strategy that provides students with the opportunity to learn and practice collaboration. Group testing is used as a modality in cooperative learning which may be useful for critical thinking skills, group processes skills, and fostering learning (4). In this method, students work in teams to prepare for instructor-created exams and take the exams first individually and next as a group. Students help each other deepen their understanding of content (5). John Dewey philosophy is at the heart of cooperative learning. Team objective structured clinical examination (TOSCE) is another type of team or group testing. Amini and colleagues used team objective structured clinical examination (TOSCE) for measuring clinical competency of teams. They reported team work and problem solving were essential for success in such exams (6). The most important advantage of collaborative learning is enhancement of learning through a decrease in anxiety arising from the exam, critical analysis of complex situations, promotion of group work, more retention, encouragement of students to be engaged in the learning process and transfer of knowledge. In this method, emotionally and intellectually supporting the students will lead to profound learning and retention of the course content (7, 8). The results of the study by Wiggs (2011) showed that the students’ performance enhanced in collaborative testing compared to individual testing. Bloom (2009) also stated that collaborative testing was a valuable educational strategy enhancing students’ learning and evaluation. Furthermore, combination of individual and collaborative testing will lead to considerably better scores. This can also enhance retention of course content. In addition, concerns about grade inflation will be adjusted using proportionally weighted scores (9).

 The need for clinicians who are able to use and apply their knowledge at clinical environments for solving patients’ problems, team work, and collaboration with other healthcare providers makes the more conspicuous use of modern student-centered techniques necessary. So far, a limited number of researches have been conducted on the merits of collaborative testing in nursing students in Iran. On the other hand, most exams are carried out traditionally with the aim of evaluation. Thus, this study aimed to determine the effect of collaborative testing on learning and retention of course content in nursing students of Shiraz University of Medical Sciences, Shiraz, Iran. 

## Methods

This quasi-experimental study was carried out on 84 baccalaureate nursing (BSN) students enrolled in the course, Medical- Surgical Nursing, GI disease section, in the spring 2013 and fall 2013 semesters. In this study, 39 students who had taken the course for the spring semester were selected as the control group. The intervention group, on the other hand, consisted of 45 students who had taken the course in the fall semester. Both the control and intervention groups were homogeneous with regards to the content, teaching method, and exam items. The students who participated in this study were fully informed about the purpose of the study. Written informed consents were also obtained from the participants prior to the exam. The control group participants took part in the first mid-term exam held individually in a multiple-choice format. The intervention group participants, however, took the test in a two-stage process. The first stage which was held individually included 25 multiple-choice items and took 25 minutes to answer. Immediately after the first testing, in the second stage, the students participated in collaborative testing in groups of five chosen randomly. They answered the questions in 20 minutes. In this stage, the students were allowed to discuss and change the answers. The content validity of exam questions was verified through a pilot study on 35 nursing students in which the difficulty and discrimination indexes were revised. To confirm the reliability of exam, we used Kuder-Richardson Formula 20 (K-R 20) and the reliability was found to be 0.80 (r=0.8).

Following the collaborative testing, the students completed a questionnaire that addressed their perceptions of collaborative examination. The items of this questionnaire were rated based on a Likert scale ranging from 1 to 5 (1: completely disagree, 2: disagree, 3: neither agree nor disagree, 4: agree, 5: completely agree). For score weighting and minimizing grade inflation in the intervention group, the total grades were calculated by summing up ⅔ of the individual testing grade and ⅓ of the collaborative testing grade (5, 9).

In order to investigate retention of course content in the intervention and control groups, in the second exam that was held individually four weeks later, in addition to answering the test items, the students were required to answer a subset of the questions of the first mid-term exam without prior announcement. However, they were assured that this subset’s score had no effects on their exam scores. Finally, the data were entered into the SPSS statistical software, version 14 and were analyzed using paired t-test and independent sample t-test. p-value<0.05 was considered as statistically significant. 

## Results

Among the 84 students who took part in this study, 60% were female (51) and 39.3% were male (33), with the mean age of 21±5.1 years. The mean scores of individual and collaborative exams in the intervention group in the fall semester were 75.35±10 and 92.40±2.40, respectively. The results of the paired t-test showed a statistically significant difference between the mean scores of collaborative and individual testing in the intervention group (p=0.001).

The control and intervention groups’ mean scores in the spring-fall semester were 71.25±15.3 and 92.40±2.40, respectively and the results of the independent sample t-test showed that this difference was statistically significant (p=0.001).

In addition to test performance, retention of course content was examined, as well. Comparison of the two groups’ mean scores of retention after four weeks is presented in Table 1. A significant difference was observed between the two groups’ mean scores after 4 weeks (p=0.001).

Overall, 98% of the students had positive views toward this educational approach. Also, this process was of interest to 97% of them because of the novelty of the procedure from the instructional point of view. According to 96% of the students, collaborative testing created and improved positive communication among the students. Moreover, 90% of the students considered this kind of testing and learning to be more effective than traditional methods. Additionally, 82% believed that they were less anxious during the exam and 78% stated that collaborative testing increased their self-confidence. 

**Table1:** Retention of course after 4 weeks (Fall-Spring2013)

**Test Grade**	**Mean±SD**	**N**	**p**
**Control group**	36.25(17.40)	32	0.001
**Intervention group**	73.80(14.5)	30	

## Discussion

The results of our study demonstrated that collaborative learning considerably enhanced learning and test performance in the intervention group compared with the control group. These findings supported those of the previous studies published in this field (4, 7, 9-11). Increased student performance has been reported when instructors create opportunities for students to become actively engaged in content and peers (8). Eastridge (2014) reported that collaborative testing incorporates approaches recommended to increase the students’ success (12). Wiggs et al. also showed that in addition to enhancing testing performance, collaborative testing could lead to development of critical thinking behaviors, group work, and decrease in anxiety arising from the exam. Active engagement in discussions is a key factor for success in the collaborative approach. Also, different factors as mediators may enhance performance in collaborative testing. The results of a prior study (13) indicated that cognitive process, interpersonal interactions, and decrease in test anxiety, as three mediating factors, played a key role in enhancing performance in collaborative testing. Therefore, enhancement of testing performance supports the concept that collaborative activities facilitate students’ learning.

The findings of the present study demonstrated that retention of course content increased considerably in the intervention group compared with the control group. In the same line, Bloom (2009) and Cortright (2003) reported that besides improving testing performance, collaborative testing would lead to enhancement of retention of course content (9, 11). This was consistent with the results of the present study. It should be noted that Bloom interpreted enhancement of the scores and long-term retention of the learned materials using cognitive theory (9).

In several studies, despite enhancement of testing performance, no statistically significant difference was found between collaborative and traditional testing regarding long-term retention of the learned material (4, 14). This can be attributed to the difference in the studies’ methodologies. Leight et al. conducted a study on 250 biology students who had been taught through an active learning method. The results showed that although the scores of group testing were higher than those of individual testing, there was no significant difference between the two groups regarding retention of course content. They stated that for interventions, such as collaborative testing, increase in retention of course content was more difficult for the students who had already experienced active learning compared to those in a lecture-based class (15).

Although the results of researches have shown that the students have a sense of responsibility towards their team and assume their own learning responsibility towards the success of the team (16), some researchers set forth grade inflation about the process of collaborative learning, stating that the results of the exam do not truly reflect the students’ individual capabilities. In collaborative testing, no statistically significant inflation has been observed compared with the groups of individual testing (13). Jensen et al. reported that the students’ grades had increased about 10% (17). In order to minimize this problem in this study, the score of the exam was calculated by summing up⅔  of the individual testing score and ⅓ of the collaborative testing score. The results of the study did not show any statistically significant inflation.

The majority of the students had positive views towards this educational strategy and recommended it for future examinations. Most of the students reported a decrease in their anxiety during the exam, which has probably had a role in enhancement of testing performance. Similar results have also been obtained in previous studies (4, 7, 10, 11).

**Limitations and recommendations**

Holding two exams simultaneously is time-consuming. Therefore, it is recommended that this procedure be used for quizzes and mid-term exams with lower volume of course subjects. Due to small sample size and doing the study in one center,it is difficult to generalize the results to other settings. Also, considering the controversy among the researchers on the effect of collaborative testing on the long-term retention of learned materials, it is suggested that further studies be carried out on larger sample sizes with more emphasis on this issue. 

## Conclusion

Exams are mainly prepared as an instrument for evaluation and little attention has been paid to their role in learning and long-term retention of learned materials. In addition to being an instrument for evaluation, collaborative testing can also be used as a useful educational strategy for promotion of learning. The results of this study showed an improvement in testing performance and enhancement of retention of course content incollaborative testing compared with the traditional method. Thus, collaborative testing, as an active learning technique and a valuable assessment method, can help nursing teachers provide the alumni with strong problem-solving and critical thinking abilities at healthcare environments. 
